# Survival of the drowsiest: the hibernating 100S ribosome in bacterial stress management

**DOI:** 10.1007/s00294-017-0796-2

**Published:** 2017-12-14

**Authors:** David W. Gohara, Mee-Ngan F. Yap

**Affiliations:** Edward A. Doisy Department of Biochemistry and Molecular Biology, Saint Louis University School of Medicine, Saint Louis, MO 63104 USA

**Keywords:** Ribosome, Translation, HPF, HflX, Stress response

## Abstract

In response to nutrient deprivation and environmental insults, bacteria conjoin two copies of non-translating 70S ribosomes that form the translationally inactive 100S dimer. This widespread phenomenon is believed to prevent ribosome turnover and serves as a reservoir that, when conditions become favorable, allows the hibernating ribosomes to be disassembled and recycled for translation. New structural studies have revealed two distinct mechanisms for dimerizing 70S ribosomes, but the molecular basis of the disassembly process is still in its infancy. Many details regarding the sequence of dimerization-dissociation events with respect to the binding and departure of the hibernation factor and its antagonizing disassembly factor remain unclear.

## Introduction

**Fig. 1:**
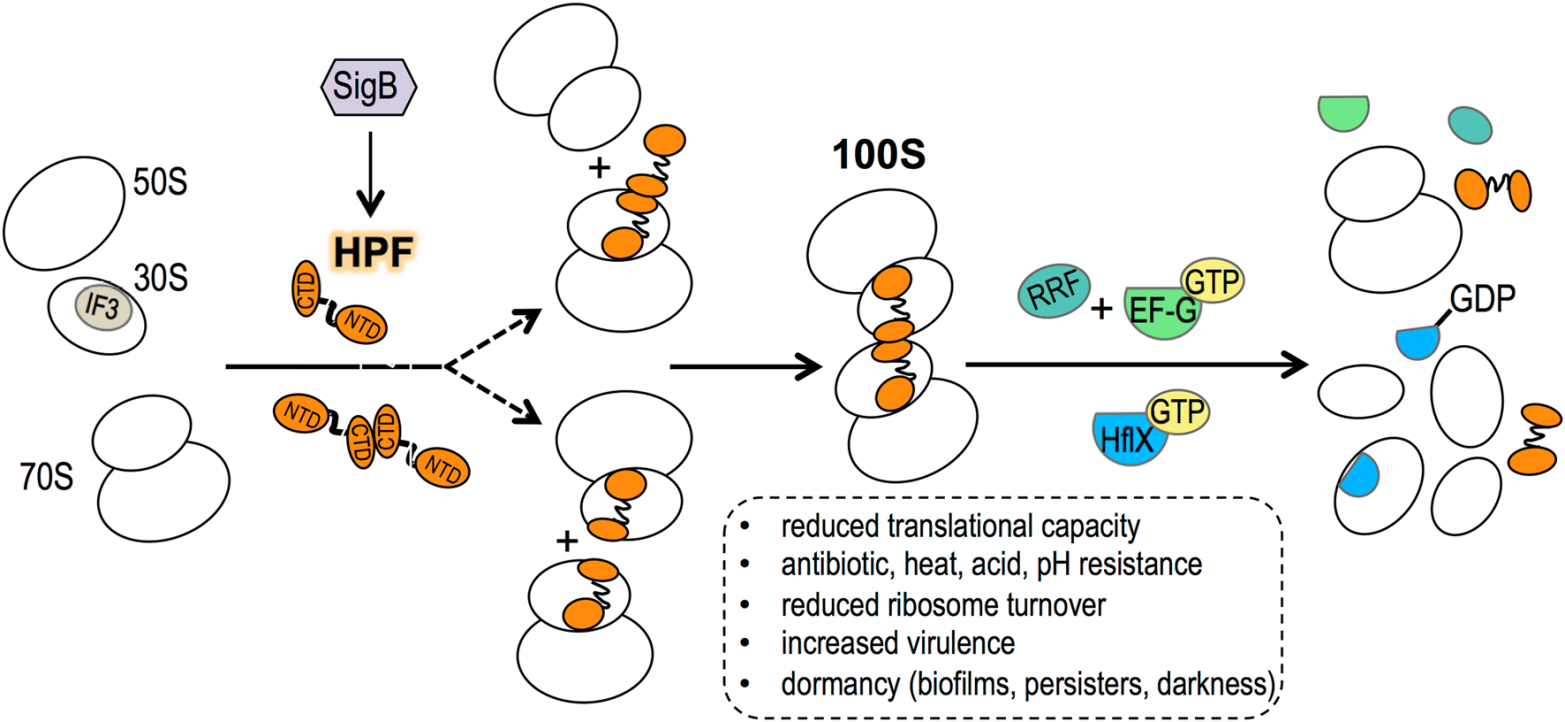
A simplified series of events on the biogenesis of the *S. aureus* 100S ribosome. The post-termination vacant ribosomes and/-or newly synthesized ribosomes could serve as the precursors of the 70S dimerization. The IF3 initiation factor prevents subunit joining but is unable to saturate all of the 30S subunits due to low cellular concentrations. The general stress response sigma factor B (SigB) activates *hpflong* expression. *S. aureus* HPF_long_ is a basic protein made of 190 amino acids that consists of the N-terminal domain (NTD) and C-terminal domain (CTD) connected by an unstructured region. It is unclear whether the HPF_long_ binds to the 70S ribosome as a monomer or a dimer. The CTD-HPF_long_ dimerization provides the primary binding platform, while the uS2-h26 and CTD-h40 interactions play secondary roles. The hibernating ribosomes enable cells to survive under various harsh environments. The recycling factor pair (EF-G and RRF) and the GTPase HflX presumably dissociate the 100S ribosomes into 70S or 30S/50S subunits

The bacterial 100S ribosome is a homodimeric particle of 70S complexes that are individually composed of 30S and 50S ribosomal subunits. The 70S pair dimerizes end-to-end via the joining of the 30S–30S ribosomal subunits (Fig. 1). The 100S ribosome was first discovered in *Escherichia coli* in the late 1950s (Huxley and Zubay 1960; Tissieres and Watson 1958). It was originally thought to undergo passive aggregation of the 70S ribosome until recently, when its physiological significance and the active participants of 70S dimerization became widely appreciated (Yoshida and Wada 2014). While the 70S complexes can either be translationally silent or competent upon binding to mRNA templates, 100S ribosomes are devoid of translational activity and often accumulate during the late stationary growth phase; hence, the inactive 70S and 100S complexes were dubbed “hibernating ribosomes”.

Ribosome hibernation is critical for bacterial adaptation to various environments. 100S ribosome-proficient cells exhibit a greater survival rate both in vitro and inside animal hosts by 2–3 orders of magnitude. Under standard laboratory conditions, the stability of the *E. coli* 100S ribosome is reduced in the presence of low magnesium, high salt, extreme pH (Tissieres and Watson 1958), and nutrient replenishment in culture (Aiso et al. 2005; Wada et al. 1990). The dissociation process of the 100S ribosome is poorly understood, although it is believed to allow reentry of mature ribosomes into the active translation pool, thereby bypassing the need of synthesizing new ribosomes that are energetically costly. By contrast, dimerization of 70S ribosomes has been investigated further in several bacterial species. The mammalian equivalent of the bacterial 100S ribosome, a dimer of 80S monomers (110S complex), has been observed in nutrient-deprived cancer cells, but the dimerizing factor has yet to be identified (Krokowski et al. 2011). Most inactive ribosomes in eukaryotes exist as non-translating 80S monomers (van den Elzen et al. 2014). This review will focus on recent findings that have advanced our understanding of the mechanism of 70S dimerization and the diverse roles of the 100S ribosome in bacterial survival (Fig. 1). Finally, we also discuss outstanding questions that have emerged from recent structural, biochemical and genetic studies.

## The many flavors of ribosome hibernation factors

In the γ-proteobacterium *E. coli*, the ribosome modulation factor (RMF_Ec_) and hibernation promoting factor (HPF_Ec,_ formerly YhbH) cooperatively stimulate the dimerization of 70S monomers, first by RMF_Ec_-induced formation of the 90S particles, followed by HPF_Ec_-mediated stabilization of the dimer. The third protein, YfiA_Ec_ (also known as pY or RaiA), is a paralog of HPF_Ec_ that silences the 70S monomer and antagonizes 70S dimerization. YfiA inhibits protein biosynthesis by sterically occluding the binding of tRNAs to the decoding sites of the 30S subunit (Agafonov and Spirin 2004; Polikanov et al. 2012; Ueta et al. 2005; Vila-Sanjurjo et al. 2004). YfiA is absent outside of the γ-proteobacteria but is functionally homologous to PSRP1 in plant chloroplasts (Bieri et al. 2017; Sharma et al. 2010).

**Fig. 2:**
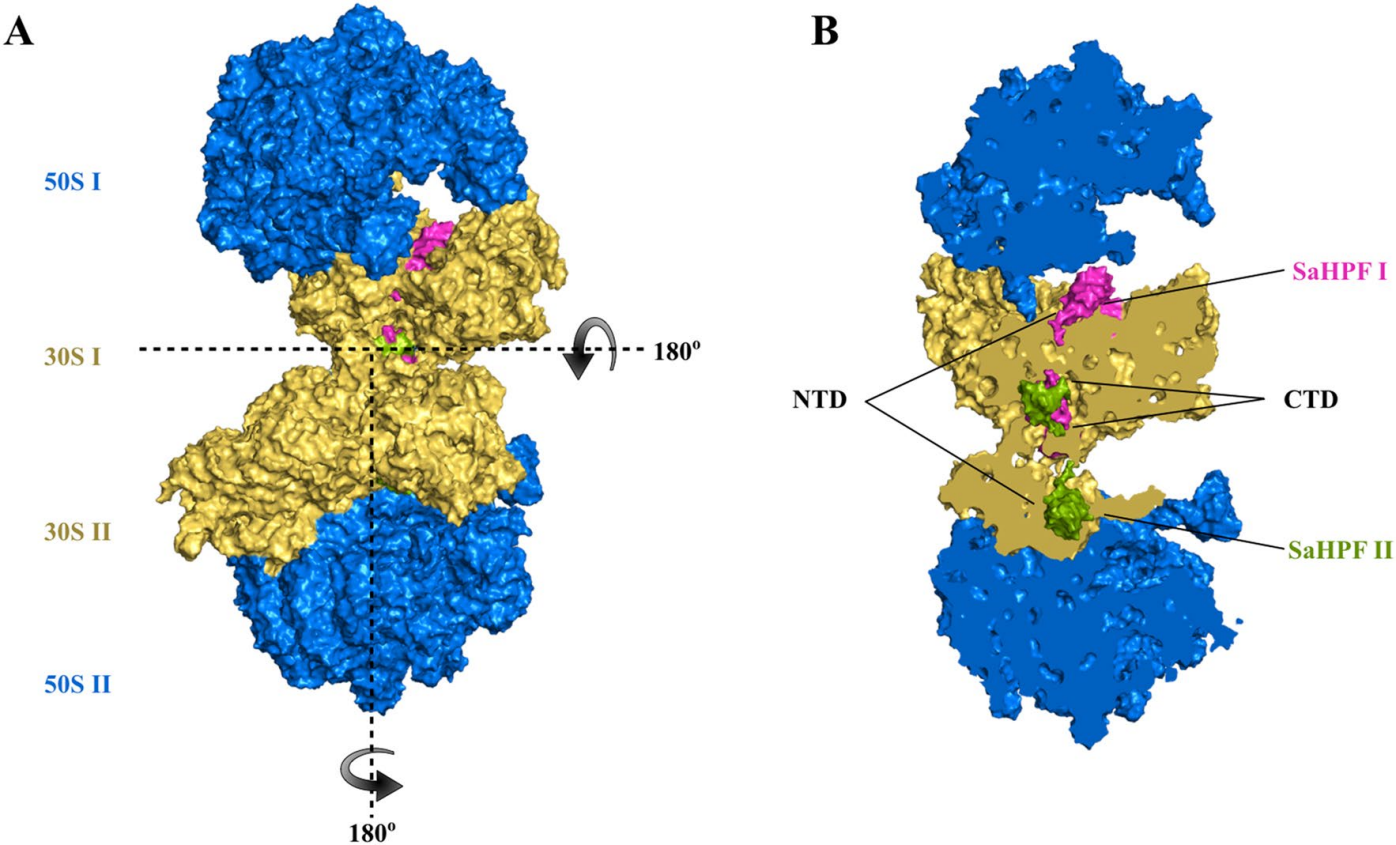
Overview of the native *S. aureus* 100S ribosome. The 100S particle consists of two 70S ribosomes, each consisting of a 50S (blue) and 30S (gold) subunit. The two 70S monomers form an interface via the 30S subunits and are tethered together by two *S. aureus* hibernation-promoting factor (SaHPF) molecules (magenta and green). **a** Relative to the top 70S ribosome, the second 70S ribosome is oriented by 180° rotations along the horizontal and vertical axes of the page. **b** Same view as **a**. The 100S particle has been sliced to better show the location and interaction of the two SaHPF molecules. Each SaHPF molecule is composed of an N-terminal domain (NTD; residues 1–95) and a C-terminal domain (CTD; residues 130–190) connected by a flexible linker. The CTD of each SaHPF molecule interact at the 30S/30S interface

The majority of bacteria carry only one long form of HPF (HPF_long_, ~ 200 amino acids long) that is twice the size of HPF_Ec_. The bipartite HPF_long_ proteins consist of the translational silencing N-terminal domain (NTD) and a dimerizing C-terminal domain (CTD) linked by an unstructured region composed of 16–62 residues (Franken et al. 2017) (Figs. 1, 2). Intriguingly, the temporal abundance of the 100S ribosome varies significantly across species. The picture that has now emerged is that *E. coli* and *Pseudomonas aeruginosa*, which carry both RMF_Ec_ and HPF_Ec_ homologs, produce 100S ribosomes during the stationary phase (Ueta et al. 2013; Wada 1998; Williamson et al. 2012). By contrast, 100S ribosomes are constitutively produced in HPF_long_-harboring bacteria, including *Staphylococcus aureus, Bacillus subtilis*, and *Lactococcus lactis* (with the exception of cyanobacteria), from the lag log phase through the late stationary phase (Davis et al. 2014; Ueta et al. 2010, 2013). The stability of the *hpf* mRNA and protein, leaky transcriptional activation of the hibernation factors, and levels of 100S disassembly factor(s) may account for the differences in temporal production (see below).

## Functional consequences of the loss of 100S ribosomes

Null mutants of *rmf* and *hpf* are phenotypically diverse from Proteobacteria to Fermicutes, suggesting the existence of a species-specific role of the 100S ribosome. An *E. coli rmf*_*Ec*_ mutant is susceptible to nutrient limitation, acid and heat stress (El-Sharoud and Niven 2007; Niven 2004; Yamagishi et al. 1993). *Listeria monocytogenes* lacking *hpf*_*long*_ are more than 20 times less virulent in a mouse model of infection and are more sensitive to killing with aminoglycosides (Kline et al. 2015; McKay and Portnoy 2015). Membrane damage may contribute to antibiotic sensitivity, similar to the loss of membrane integrity observed in a *P. aeruginosa* Δ*rmf* mutant (Williamson et al. 2012). The *S. aureus hpf*_*long*_ null or dimerization-defective strain has an extremely low survival rate in long-term culture (Basu and Yap 2016) and under acute heat exposure (Matzov et al. 2017). *hpf* knockouts of *L. lactis, B. subtilis, and P. aeruginosa* fail to resuscitate from starvation (Akanuma et al. 2016; Akiyama et al. 2017; Beckert et al. 2017; Puri et al. 2014). The *Synechococcus elongatus* Δ*hpf* mutant undergoes translational derepression (Hood et al. 2016) analogous to a *S. aureus* Δ*hpf* mutant (Basu and Yap 2016). Bacteria lacking the 100S ribosome are prone to cell death, a feature that is accompanied by massive ribosome degradation (Akiyama et al. 2017; Basu and Yap 2016; Shcherbakova et al. 2015). The causal relationship between ribosome integrity and the formation of the 100S ribosome is unclear. The structure of the 100S ribosome may be more resistant to RNase, and the binding of hibernation factors may compete with the RNase target sites. It is also possible that the expression of specific factors involving in the ribosome decay pathway (Redder 2016) is compromised in the *rmf* and *hpf* mutants.

In general, the phenotypes described above are manifested during slow growth, such as in aging and dormant cells, and intracellular growth inside the host cells, which are normally characterized by metabolic arrest and translational dormancy that restrict energy consumption (Dai et al. 2016). Consistent with this observation, the levels of HPF_long_ or RMF are substantially elevated in a murine pneumonia model (Michalik et al. 2017), in starved cells (Aiso et al. 2005; Sanchuki et al. 2017), in biofilms (Williamson et al. 2012), and in non-replicating persisters (Tkachenko et al. 2017). Moreover, HPF_long_ reduces translational efficiency of a subset of genes in vivo (Basu and Yap 2016; Hood et al. 2016) and in cell-free translation assays (Basu and Yap 2016; Ueta et al. 2008, 2013), presumably because 70S dimerization titrates functional ribosomes away from translational initiation. Paradoxically, deficiency of HPF_long_ may be favorable under certain antibiotic stress conditions, as reported in *Synechocystis* sp. and *Streptomyces venezuelae* (Galmozzi et al. 2016; Jones et al. 2014).

## Mechanistic differences of 70S dimerization

Four recent cryo-EM maps of the HPF_long_-bound 100S ribosome have revealed a surprising mechanism of 70S dimerization in three fermicutes (*S. aureus, B. subtilis* and *L. lactis)* that is fundamentally distinct from that of the *E. coli* counterpart. In all three species, the translation-inactivating NTD of HPF_long_ adopts a βαβββα fold and occupies the tRNA- and mRNA-binding sites of the 30S subunit that is virtually superimposable on the HPF_Ec_. In all cases, the CTD-HPF_long_ on one copy of the 70S monomer slightly extends outside of the solvent-accessible face of the 30S subunit and directly interacts with another CTD-HPF_long_ that is tethered on the opposite copy of the 70S monomer (Figs. 1, 2). Aside from the primary CTD–CTD contact in *S. aureus*, secondary interactions between the ribosomal protein uS2 and rRNA h26 and CTD-h40 further stabilize the dimer interface (Matzov et al. 2017). Similar interactions have been observed in *L. lactis* (Franken et al. 2017). HPF_long_ in *S. aureus* also induces a 5° swiveling of the 30S head domain, but this movement was not seen in other cryo-EM maps. Notably, the uS2-h26 and CTD-h40 interactions are absent in the second *S. aureus* cryo-EM model reported by Khusainov et al. and, instead, are replaced by an h26-h26 interaction between the two 30S subunits (Khusainov et al. 2017). The discrepancies may be attributed to different approaches in the preparation of 100S ribosomes. One was purified directly from cell culture whereas the other was reconstituted in vitro using a recombinant HPF_long_ protein. In *Bacillus subtilis*, the secondary contacts are established between the uS2-bS18 and uS2-h26 pairs on the opposing 30S subunits (Beckert et al. 2017). Despite these differences, a convergent rule emerges from these studies is that the physical CTD–CTD interaction is crucial for HPF_long_-induced dimerization.

**Fig. 3:**
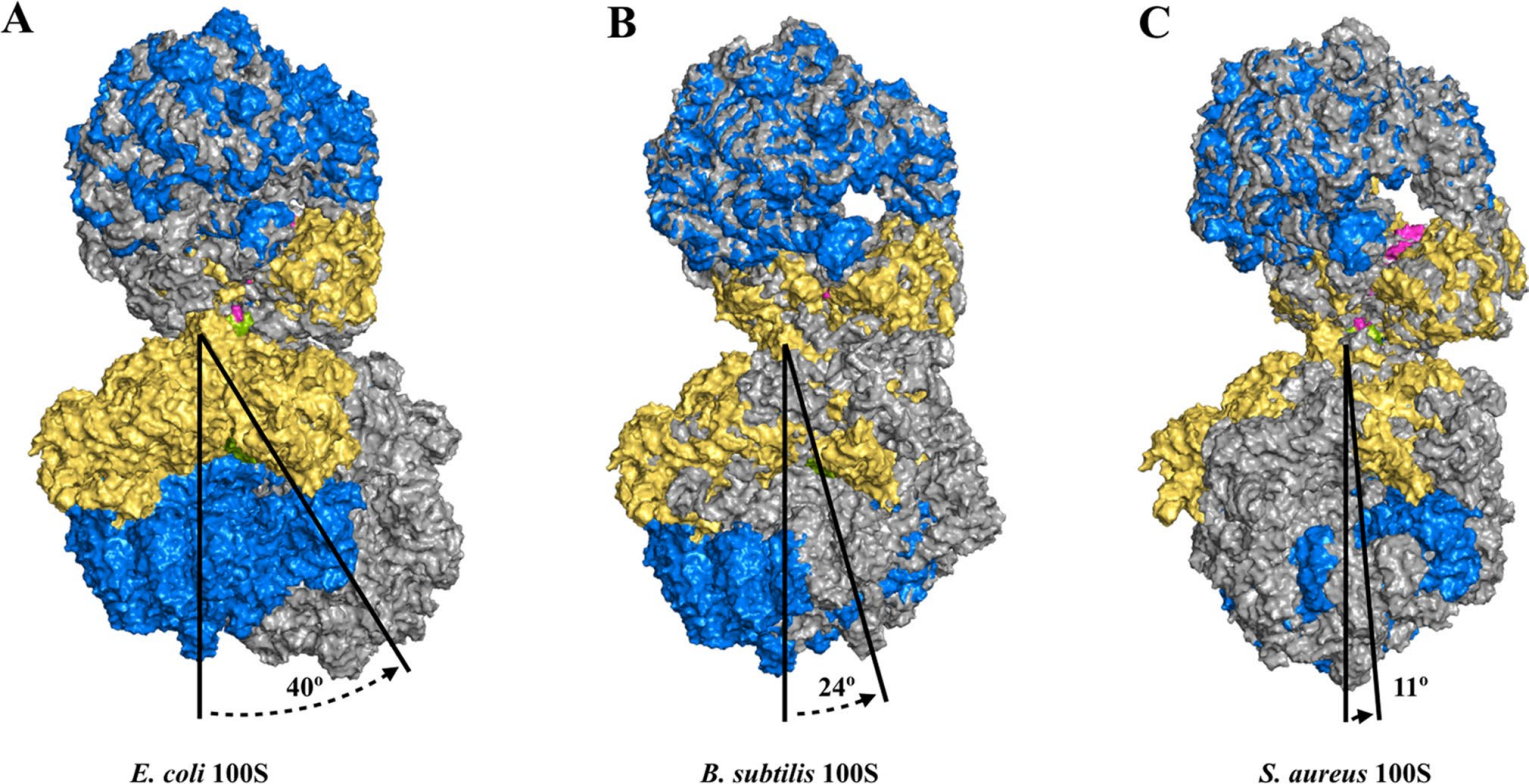
Comparison of multiple 100S particle orientations with respect to the *S. aureus* 100S ribosome. The 100S particle structure from *S. aureus* (PDB 5NG8) is colored and oriented as shown in Fig. 2. Cryo-EM maps for *E. coli* (**a** EMD-5174), *B. subtilis* (**b** EMD-3664) and a second structure determination for *S. aureus* (**c** EMD-3638) were downloaded from the Electron Microscopy DataBank (EMDB). For each map, atomic coordinate files were obtained from the RCSB. No corresponding model was available for the *E. coli* cryo-EM map. Therefore, the 5DFE structure was used instead. Deposited coordinate files for 5NJT and 5ND8 were used for *B. subtilis* and *S. aureus*, respectively. With the exception of 5NG8, 100S particles were generated from the 70S ribosome coordinates. For each structure, a second 70S monomer was generated and manually docked (translated and rotated) into their corresponding electron density maps using PyMOL v1.8.6 (Schrödinger). The overall goodness of fit was qualitatively assessed by visual inspection. In the case of *E. coli*, the electron density map contained only the first 70S monomer and a portion of the density for the second 30S subunit. The partial density for the second 30S subunit was used to align and orient the entire second 70S monomer relative to the first. Following construction of the complete 100S structures, each structure was superpositioned onto 5NG8 using the top 70S ribosome and by maintaining the relative orientation of the second 70S ribosome as defined by the cryo-EM maps prior to superpositioning. Using the bottom 70S ribosome from 5NG8 as a reference (blue and gold), the relative orientation for *E. coli, B. subtilis* and *S. aureus* (all in grey), represent approximate 40, 24 and 11 degree counterclockwise rotations, respectively, along the plane of the page

By contrast, *E. coli* RMF_Ec_ allosterically promotes 70S dimerization without any contact between the two RMF_Ec_ molecules. Instead, RMF_Ec_ induces conformational changes in the 30S subunit and, in turn, widens the dimer interface to accommodate interactions that comprise of rRNA h39, and the uS2, uS3, and uS5 ribosomal proteins (Kato et al. 2010; Ortiz et al. 2010; Polikanov et al. 2012). RMF_Ec_ is mapped to a site that blocks the binding of the 30S subunit to the mRNA Shine–Dalgarno (SD) sequence distantly away from where the CTD-HPF_long_ binds. The differences in the 30S binding mode affect the overall dimeric architectures. A comparison of various 3D maps of 100S ribosomes demonstrates that the orientation of the native *S. aureus* 100S ribosome (Matzov et al. 2017) deviates from that of *E. coli, B. subtilis*, and *S. aureus* (Khusainov et al. 2017) by approximately 40°, 24°, and 11° counterclockwise rotations, respectively (Fig. 3).

## Disassembly of the 100S ribosome

Dissociation of the 100S ribosome into 70S or 30S and 50S subunits may provide renewable ribosomes for restarting translation. This process likely involves an active mechanism to dislodge hibernation factors from the ribosome. Recent biochemical and genetic studies have demonstrated that an evolutionarily conserved GTPase known as HflX disassembles both the *S. aureus* vacant 70S and 100S ribosomes, but only the dissociation of the 100S ribosome requires GTP hydrolysis (Basu and Yap 2017). *Escherichia coli* HflX binds to the peptidyltransferase center of the 50S subunit (Zhang et al. 2015). Thus, the *S. aureus* HflX may bind to similar regions and split the 100S ribosome by disrupting the intersubunit bridges of the 70S ribosome, given that breaking just one of the twelve intersubunit bridges (uS13-bL31 B1b bridge) could collapse subunit joining and reduce the formation of 100S (Ueta et al. 2017). HflX is likely not the only disassembly candidate because an Δ*hflX* knockout exhibits a modest phenotype (Basu and Yap 2017), and the *E. coli* ribosome recycling factor (RRF), in conjunction with EF-G, is able to partially rescue the growth defect of an *E. coli* Δ*hflX* mutant (Zhang et al. 2015). Other canonical translation factors such as IF3, in addition to RRF and EF-G, have been implicated in counteracting the formation of the *E. coli* 100S complex and the hibernating 70S ribosome in chloroplasts (Sharma et al. 2010; Yoshida et al. 2009).

## Regulation of RMF and HPF

Small signaling molecules positively control the levels of hibernation factors under specific stress conditions. *Escherichia coli rmf*_*Ec*_ is a member of the polyamine and cAMP modulons (Shimada et al. 2013; Tkachenko et al. 2017). The stationary phase-specific stringent response alarmore (p)ppGpp up-regulates the transcription of *E. coli rmf*_*Ec*_ and *hpf*_*long*_ of *B. subtilis* and *S. elongates*. (Hood et al. 2016; Tagami et al. 2012; Terui et al. 2010). Although ppGpp does not appear to directly influence the expression level of HflX GTPase, ppGpp binds and inhibits the 100S splitting activity of HflX (Basu and Yap 2017; Corrigan et al. 2016). The intracellular ppGpp concentration is inversely correlated with GTP synthesis. This raises the possibility that ppGpp-GTP homeostasis may play an intricate role in regulating the expression and function of HPF and HflX. Collectively, the transcription of *rmf* and *hpf* are sensitive to environmental stimuli. For instance, nitrogen- and carbon-starvation increase *rmf*_*Ec*_ expression whereas thermal stress or darkness strongly induces *hpf*_*long*_ transcription.

In Fermicutes and cyanobacteria, *hpf*_*long*_ is the direct target of a stress-responsive alternate sigma factor SigB whose highly conserved binding motif is found at the 5′-UTR of the *hpf* operon. *B. subtilis hpf*_*long*_ is also under positive control of the sporulation regulator SigH (Akanuma et al. 2016; Basu and Yap 2017; Galmozzi et al. 2016; Kline et al. 2015). Deletion of *sigB* in *S. aureus* does not completely abolish the synthesis of HPF_long_. Additional putative binding motifs of housekeeping SigA (or σ^70^) and global regulator CodY are detected upstream of the *hpf*_*long*_ (Majerczyk et al. 2010; Waters et al. 2016). The additive effect of multiple regulatory activators on *S. aureus hpf*_*long*_ provides an explanation for the constitutive production of *hpf*_*long*_ and the 100S ribosome (Davis et al. 2014; Majerczyk et al. 2010; Ueta et al. 2010; Waters et al. 2016). Alternately, *S. aureus hpf*_*long*_ may be ill regulated due to the absence of a cognate repressor or long-lived transcript and protein. The turnover of *S. aureus* HPF_long_ is relatively slow even when the ribosome concentration drops substantially after 4 days in culture (Basu and Yap 2016). Furthermore, the mRNA structure may prolong the transcript half-life and, in turn, determines the biosynthesis of the hibernation factors (Redder 2016). Due to the 5′-UTR hairpin structure, the *rmf*_*Ec*_ mRNA has an unusually long half-life during the early exponential phase (24 min *versus* the average *E. coli* transcripts of 1–2 min) and 120 min during the stationary phase (Aiso et al. 2005).

## Outlook

The formation of the 100S ribosome is one of the most important survival strategies in bacteria to help cope with various environmental stressors. Recent structural and genetic studies have opened new avenues of research to investigate structure–function relationships and the biological role of the 100S ribosome. Despite several recent advances, many outstanding questions remain to be addressed to obtain a more complete picture of ribosome hibernation (Fig. 1):

1. At which stage of translation does dimerization occur? Surprisingly, overexpression of HPF_long_ does not significantly retard cell growth, and an *hpf* null mutant only modestly increases global translation (Basu and Yap 2017). This finding raises the possibility that ribosomes are usually produced in excess during exponential growth and that the translation capacity is not significantly compromised when 20–40% of the ribosomes are converted to the silent 100S ribosome. Thus, the newly synthesized free ribosomes that have yet to engage in translation may be the targets of HPF_long_. Another possibility is that post-termination 70S ribosomes do not undergo subunit splitting (Chen et al. 2017; Orelle et al. 2015; Yamamoto et al. 2016) and instead serve as precursors for dimerization. The latter may occur more frequently during transition from log phase to stationary phase when the ribosomes disproportionally outnumber mRNA molecules.
2. The precise role of dimerization in unclear. Bacteria have evolved two disparate mechanisms of 70S dimerization, yet they share an overlapping dimer interface. This finding suggests that these regions are protected and may be vulnerable to nucleolytic attacks and thermal insults. Alternately, these regions may be a hotspot of unintended molecular interactions in the crowded cytoplasm. Bacterial transcription and translation are coupled, and RNA polymerase (RNAP) may bind to the 70S dimer interface (Fan et al. 2017; Kohler et al. 2017). Thus, dimerization may simply prevent spurious interactions with the RNAP.
3. The mechanistic details of dimerization and translation inactivation are poorly defined. Truncated CTD-HPF_long_ variants self-dimerize in solution and are sufficient to induce the formation of the 100S ribosome in vivo, albeit less effectively than the full-length HPF_long_ (Beckert et al. 2017; Matzov et al. 2017; Puri et al. 2014). 70S dimerization may be initiated through one of two pathways (or both). First, a free HPF-dimer binds to one copy of the 70S monomer and seizes the second 70S monomer. Second, each free HPF monomer binds to individual 70S monomers, and dimerization occurs when the CTDs make contacts with each other. Discerning these scenarios will require time-resolved kinetic studies of 70S dimerization coupled with monitoring the oligomeric state of HPF_long_. Along the same line, it is unclear whether monomeric HPF_long_ and dimeric HPF_long_ on a single 70S monomer equally achieve translational repression compared to the mature 100S complex.
4. How is the 100S ribosome remobilized into the active translation pool? Does the RRF/EF-G pair play a more prominent role than HflX in the disassembly of the 100S ribosome? Future kinetic studies are needed to delineate the precise sequence of 100S disassembly and the hierarchical order of IF3, RRF/EF-G, and HflX as well as to monitor the fates of HflX and HPF upon dissociation. Furthermore, HPF_long_ appears to be abundant over a long period of time. Therefore, it is unclear whether proteolysis occurs following the detachment of HPF_long_ from the ribosome or whether an unknown factor sequesters free HPF_long_ away from re-dimerizing the 70S ribosomes.
